# Dataset of first appearances of the scholarly bibliographic references on Wikipedia articles

**DOI:** 10.1038/s41597-022-01190-z

**Published:** 2022-03-14

**Authors:** Jiro Kikkawa, Masao Takaku, Fuyuki Yoshikane

**Affiliations:** Faculty of Library, Information and Media Science, University of Tsukuba, 305-8550 Ibaraki, Japan

**Keywords:** Scientific community, Social sciences

## Abstract

Referencing scholarly documents as information sources on Wikipedia is important because it supports or improves the quality of Wikipedia content. Several studies have been conducted regarding scholarly references on Wikipedia; however, little is known of the editors and their edits contributing to add the scholarly references on Wikipedia. In this study, we develop a methodology to detect the oldest scholarly reference added to Wikipedia articles by which a certain paper is uniquely identifiable as the “first appearance of the scholarly reference.” We identified the first appearances of 923,894 scholarly references (611,119 unique DOIs) in 180,795 unique pages on English Wikipedia as of March 1, 2017 and stored them in the dataset. Moreover, we assessed the precision of the dataset, which was highly precise regardless of the research field. Finally, we demonstrate the potential of our dataset. This dataset is unique and attracts those who are interested in how the scholarly references on Wikipedia grew and which editors added them.

## Abstract (summary)

**Table:** 

Measurement(s)	Publication • Scientific Publication • Publication Identifier • Digital Object Identifier (DOI)
Technology Type(s)	documenting • digital curation

## Background & Summary

Along with the digitization of scholarly communication, numerous scholarly documents have been referenced and used on the Web. One of the changes arising from the development and dissemination of scholarly information infrastructures on the Web is the utilization of scholarly documents by various people and communities, including readers other than traditional ones such as researchers and specialists. As such an example, there are many references and accesses to scholarly documents via Wikipedia. In particular, according to Crossref, which assigns Digital Object Identifiers (DOIs) to scholarly documents massively, Wikipedia is one of the largest referrers of Crossref DOIs as of 2015^1^.

Wikipedia is a free online encyclopedia that anyone can edit, and it has been one of the most visited websites in the world. However, owing to its collaborative nature, much criticism and discussion have emerged since its start with regard to the accuracy and reliability of its contents. Three core content policies exist in Wikipedia: “verifiability,” “neutral point of view,” and “no original research.” Referencing scholarly documents as information sources on Wikipedia complements these policies, as these cited sources support or improve the quality of Wikipedia content.

Several studies have been conducted regarding scholarly bibliographic references on Wikipedia; however, most of them have focused on the scholarly document itself^2–6^. The methodologies in previous studies used to identify the scholarly references on Wikipedia can be classified as follows: (1) extracting them from either page texts (Wikitexts) or Wikipedia external links^2–4^, (2) detecting the pages contain them by using web search engines^5^, and (3) analyzing usage log data^6^. However, these methods cannot identify the first appearance for each reference on the page, that is, when it was added and by whom. Hence, little is known of the editors and their contributions to the addition of scholarly references to Wikipedia.

**Fig. 1:**
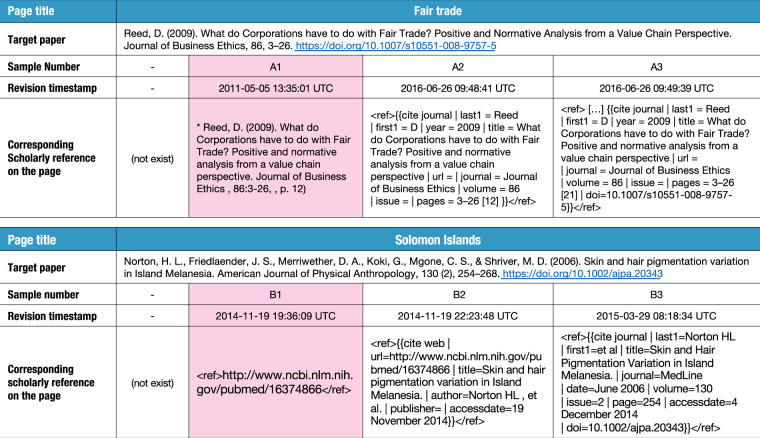
Example of the scholarly references on English Wikipedia. The first appearances for the target papers on the pages “Fair trade” and “Solomon Islands” are colored in pink (A1 and B1, respectively). There are no corresponding references in previous revisions of the pages. As for the former, an editor had added the corresponding scholarly reference including the author name, published year, paper title, and journal name to the page on A1, then another editor modified its format according to the citation template on A2, and DOI was added on A3. As for the latter, initially an editor added just the URI with PubMed ID (PMID) on B1, then the paper title and author names for the paper were added along with modification of the format according to the citation template on B2. Additional information including DOI was added on B3.

In this study, we define the term “first appearance of the scholarly reference” as the oldest scholarly reference added to Wikipedia articles by which a certain paper is uniquely identifiable. We did not consider the roles of each reference. That is, for example, references as evidence for a certain part of content of the page, those just mentioning the paper, and those listed in further readings are not distinguished in this study. If there are multiple references corresponding to the same paper on the same page, the oldest one is treated as the first appearance. Fig. 1 illustrates examples of scholarly references on English Wikipedia. The most difficult part is that the scholarly reference at the time of its first appearance is composed of insufficient or incomplete information, and more detailed information is added in later revisions.

A dataset by Halfaker *et al*.^7^ captured the revisions where the scholarly citations were added to the pages on the 298 Wikipedia languages editions. In particular, they extracted identifiers such as DOI, arXiv, ISSN, and ISBN on the page. However, the method used to build their dataset cannot detect the first appearances for cases such as A1 in Fig. 1.

Considering this background, the authors have proposed methods to identify the first appearances of the scholarly references on Wikipedia by using the paper titles and their identifiers and built a dataset of first appearances of the scholarly bibliographic references on English Wikipedia articles^8^. We then evaluated the precision for detecting the first appearance, which was 93.3% as a whole and exceeded 90% in 20 out of 22 research fields^8^. Thus, our methods enable the identification of the first appearance with high precision, regardless of the research field. The dataset we built through the study is unique and interesting for those who are concerned with, e.g., how the number of scholarly references grows on Wikipedia, or which editors are adding them. It would be especially valuable for researchers in fields such as scientometrics. For instance, we conducted a time-series analysis using this dataset and revealed the trends and characteristics of adding scholarly references to Wikipedia^9^.

In this paper, we provide an overview of the workflow for building the dataset above. In addition, we performed some analyses using the dataset.

## Methods

**Fig. 2:**
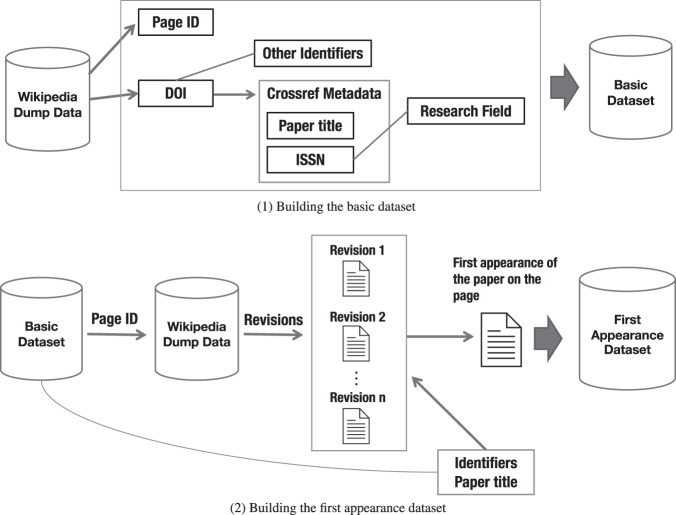
Data creation workflows.

Fig. 2 illustrates the data creation workflow in this study. There are two main parts: (1) building the basic dataset and (2) building the first appearance dataset. Hereafter, we provide an overview of each step in these workflows.

### Building the basic dataset

This process is comprised of the following 5 steps:

**Step1-1**. We extracted DOI links referenced in main namespace pages along with their page IDs and page titles on English Wikipedia as of March 1, 2017, using Wikipedia dump files^10,11^. We used SQL format files “enwiki-20170301-externallinks.sql.gz,” “enwiki-20170301-iwlinks.sql.gz,” and “enwiki-20170301-page.sql.gz.” The extraction conditions were the same as those used by Kikkawa *et al*.^4^.

**Step1-2**. We excluded non-Crossref DOIs to remove non-scholarly contents, and obtained Crossref metadata^12^ for each DOI via the Crossref REST API^13^. Additionally, we excluded non-individual contents such as an entire book or a set of scholarly articles based on values on the key “type” on Crossref metadata. That is, we removed the items whose values of the type are “book,” “journal-volume,” or “journal-issue” and limited the target to ones whose values of the type are either “journal-article,” “book-chapter,” or “proceedings-article.”

**Step1-3**. We obtained other identifiers such as PubMed (PMID & PMCID) and Bibcode corresponding to each DOI via the following Web APIs: Entrez Programming Utilities^14^ and Abstract Links^15^. Subsequently, we associated these identifiers with each DOI.

**Step1-4**. We associated the research fields with each DOI based on the ESI master journal list^16^, as of August 2017, provided by the InCites Essential Science Indicators^17^. This list represents journal names, research fields, and ISSN numbers. We converted this data into pairs of ISSN numbers and DOIs and then identified the research fields for each DOI. The relationships between a single DOI and research fields range from one to three.

**Step1-5**. We stored page IDs, page titles, DOIs, and other identifiers; Crossref metadata; and research fields for each reference as the basic dataset.

Steps 1-2/1-4 and Step 1-3 are the same as Kikkawa *et al*.^8,18^, respectively. In Step 1-2, each DOI was checked by using Which RA?^19^ and removed items as errors where the result was either “Invalid DOI,” “DOI does not exist,” “Error,” or “Unknown.” After removing errors, unique DOI names were 675,798 and 99.7% of them were Crossref DOI. Since non-Crossref DOIs were only 0.3%, excluding non-Crossref DOI has little effect on the coverage of the dataset.

### Building the first appearances dataset

This process is comprised of the following 4 steps:

**Step2-1**. We extracted all revision histories corresponding to page IDs in the basic dataset, together with page texts. In particular, we used XML format files “enwiki-20170301-pages-meta-history*.xml-*.bz2” in English Wikipedia dump files as of March 1, 2017^10,11^.

**Step2-2**. We extracted identifiers and paper titles from the basic dataset, and detected the candidates of the first appearance for each scholarly reference on the page when any of the following conditions were applied. (1) One or more identifiers obtained through Step 1-3 are included in the page text. (2) Either the full title of the paper or the first five words of the title is included in the page text. As for the reason why the first five words of the paper title were applied, we will describe it in the technical validation section. (3) The similarity score based on the edit distance between the two paper titles from the basic dataset and from the extracted citation on the page is equal to or lower than the given threshold. In particular, the similarity score is the Levenshtein distance between the two titles divided by the length of the longer titles of them. When the multiple revisions were detected by the conditions (1), (2), or (3), we selected the oldest revision among them as the first appearance.

**Step2-3**. We classified the editor of the revision into “User,” “Bot,” or “IP.” User refers to human editors among registered editors. Bot refers to non-human editors among registered editors. IP refers to non-registered editors. As Geiger & Halfaker^20^ have pointed out, it is a complex task to identify strictly whether each editor is Bot or not. In this study, to enable to focusing scholarly article additions by the human editors, we defined the Bot editors as non-human editors adding numerous scholarly references automatically, and identified them based on the following conditions: (1) the editor belongs to the Bot user group, (2) the editor belongs to the category “All Wikipedia bots^21^,” (3) the editor fulfills both his/her name includes the string “bot” in a case insensitive and showing he/she is Bot on the user page, or (4) the editor fulfills both adding equal to or more than 500 scholarly references and showing he/she is Bot on the user page. As for (3) and (4), the first author checked descriptions on user pages.

**Step2-4**. We stored the revision information for the first appearances to the final dataset along with the values in the basic dataset.

Steps 2-1 and 2-3, Steps 2-2 and 2-4 are the same as Kikkawa *et al*.^8,18^, respectively.

## Data Records

The dataset presented in this paper is available at Zenodo^22^. It includes not only the dataset of English Wikipedia as of March 1, 2017, but also English Wikipedia as of October 1, 2021, where we applied the same methodology. We show technical validations and usage notes based on the dataset as of March 1, 2017.

The data format of the dataset is JSON lines^23^, where each line is a single record. In this study, we detected the first appearance of each scholarly reference added to Wikipedia articles. If there are multiple references corresponding to the same paper on the same page, only the oldest one is collected.

Table 1 presents the structure of the dataset. There are 19 keys classified into these 3 categories: (A) bibliographic data for the referenced paper (from #1 to #12, originating from Crossref metadata), (B) information of the Wikipedia page to which the scholarly reference was added (#13 and #14), and (C) information related to the edit (e.g., who and when added it) (from #15 to #19). In Table 1, the example values are taken from the paper titled “Push or pull: an experimental study on imitation in marmosets” authored by Bugnyar & Huber, published in the journal “Animal Behaviour,” vol 54, issue 4, 1997 that is referenced on the page “Imitation.” The research field corresponding to the journal “Animal Behaviour” is “Plant & Animal Science.” This scholarly reference was added to the page at “2008–04–04 15:54:09 UTC” by the editor “Nicemr” whose type is “User.” The key “paper_published_year” is the year extracted from the key “issued” on Crossref metadata^12^, which is the earliest year of the paper published either in print or online.

**Table 1:** Overview of the dataset.

#	Key	Data type	Value	Short description
1	doi	String	“10.1006/anbe.1996.0497”	DOI corresponding to the paper
2	paper_type	String	“journal-article”	Document type of the paper
3	paper_container_title	Array (String)	[“Animal Behaviour”]	Journal title, book title, or proceedings title
4	paper_publisher	String	“Elsevier BV”	Publisher name
5	paper_title	Array (String)	[“Push or pull: an experimental study on imitation in marmosets”]	Paper title
6	paper_published_year	String	“1997”	Published year
7	paper_issue	String	“4”	Issue number
8	paper_volume	String	“54”	Volume number
9	paper_page	String	“817-831”	Page numbers
10	paper_author	Array (JSON)	[“given”:“THOMAS”, “family”:“BUGNYAR”, “sequence”:“first”, “affiliation”:[], “given”:“LUDWIG”, “family”:“HUBER”, “sequence”:“additional”, “affiliation”:[]]	Authors information consisted of given and family names, sequences (order in author names), and affiliations
11	issn	Array (String)	[“0003-3472”]	ISSN related to the paper
12	research_field	Array (String)	[“PLANT & ANIMAL SCIENCE”]	Research fields from ESI categories
13	page_id	String	“577858”	Page id
14	page_title	String	“Imitation”	Page title
15	revision_id	String	“203309031”	Revision id
16	revision_timestamp	String	“2008-04-04 15:54:09 UTC”	Revision timestamp
17	revision_comment	String	“/* Animal Behaviour */”	Revision comment (edit summary)
18	editor_name	String	“Nicemr”	Wikipedia editor’s name
19	editor_type	String	“User”	Type of the editor

## Technical Validation

### Assessment of the precision

**Fig. 3:**
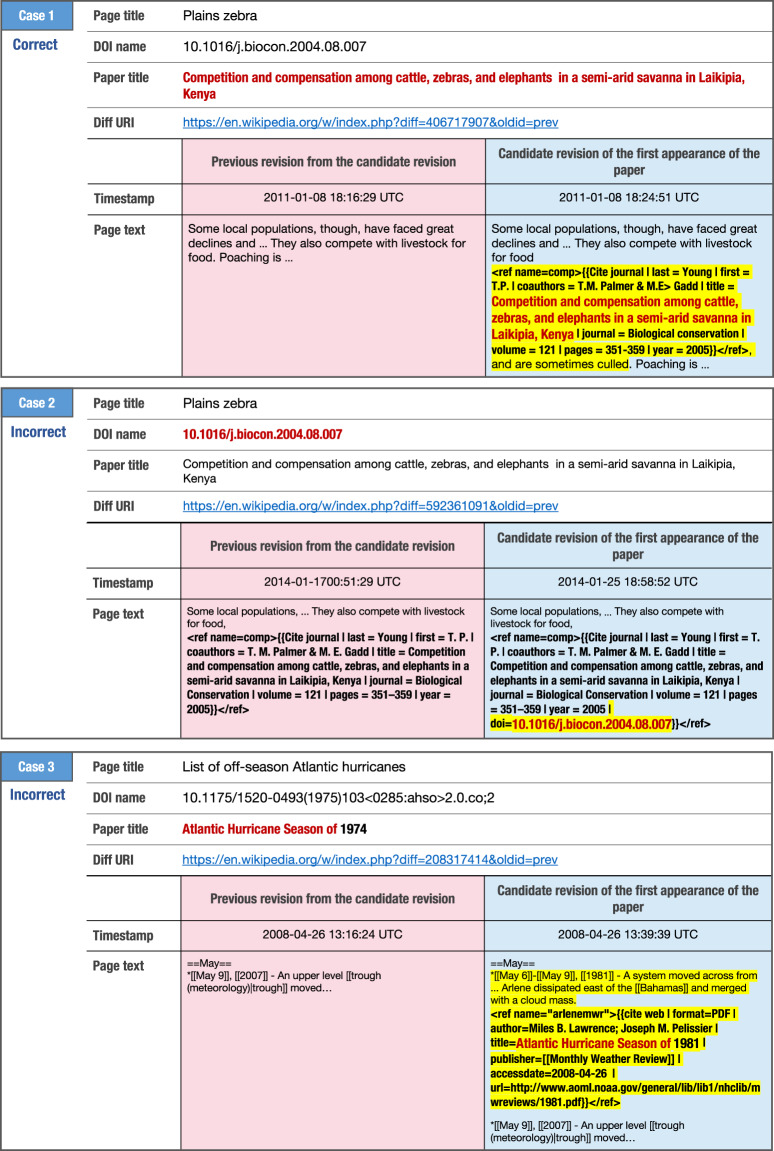
Example of the candidates for the revisions of the first appearance of scholarly references and their previous revisions. Below the case number is the judgment on the candidate for the revision of the first appearance of the paper by the first author. The box colored in light blue is the candidate for the revision of the first appearance of scholarly reference, and the box colored in pink is its previous revision. The text highlighted in yellow shows the diff from the previous revision, and the text in red is the point that fulfilled any of the conditions described in Step 2-2 on the building the first appearances dataset section.

The first appearance dataset in this study was built using the methods described above, and we evaluated the precision of the proposed methods by checking each diff^24^ between the candidate revision of the first appearance and the previous revision manually by the first author. In particular, we took random samples of 50 records for each research field, that is, 1,100 records in total from the dataset, and judged whether each of them is the first appearance. In the judgements, we confirmed changes between two revisions based on bibliographic information including author names, journal names, published years, volume and issue numbers, pages, and URI of individual scholarly references retrieved from Crossref metadata. Fig. 3 illustrates the samples of the correct and incorrect candidates of the first appearance and comparisons of the revisions.

For the cases 1 and 2 in Fig. 3, the page and the scholarly article are the same, the case 1 fulfills the conditions (2) and (3), the case 2 fulfills the condition (1) in Step 2-2 above, respectively. We judged the case 1 as the correct first appearance because the corresponding scholarly reference does not exist in the previous revision. On the other hand, we judged the case 2 as an incorrect first appearance because only the DOI name had been added to the existing reference in the candidate revision.

For the case 3 in Fig. 3, the scholarly reference added in the candidate revision is similar to the target paper. We judged it as incorrect first appearance because the papers “Atlantic Hurricane Season of 1981” and “Atlantic Hurricane Season of 1974” are different ones. Similarly, if there is no corresponding reference in the previous revision, we judged it as correct first appearance.

Based on the results, we calculated the precision for each research field using the following formula:

$$Precision=\frac{total\;number\;of\;correct\;first\;appearances}{total\;number\;of\;samples}\ast 100$$

For instance, when the number of samples judged as true first appearances was 45 in a certain research field, the precision for the field was 90.0%.

Table 2 lists the results of precision for each research field. The highest precision was 98.0% (in clinical medicine, environment/ecology, and psychiatry/psychology). On the other hand, the precisions in chemistry and physics are relatively low (86.0% and 84.0%, respectively). The reason why the precisions in chemistry and physics are relatively low lies in the conventions in these fields. In other words, scholarly references consisting of information other than the paper title and identifiers (e.g., author name, journal name, volume, issue, or pages). For example, the citation format like “Macromolecules, 2007, 40 (7), pp 2371–2379”﻿ is used in these fields. These errors are unavoidable for the methodology in this study, it would be needed to consider using additional factors such as journal names and published years to address the cases above in the future.

**Table 2:** Results of precision for identifying the first appearances on each research field based on the sample data.

#	Research field	Precision	#	Research field	Precision
1	Clinical Medicine	98.0%	12	Materials Science	94.0%
2	Environment/Ecology	98.0%	13	Neuroscience & Behavior	94.0%
3	Psychiatry/Psychology	98.0%	14	Plant & Animal Science	94.0%
4	Computer Science	96.0%	15	Microbiology	92.0%
5	Immunology	96.0%	16	Space Science	92.0%
6	Molecular Biology & Genetics	96.0%	17	Biology & Biochemistry	90.0%
7	Multidisciplinary	96.0%	18	Engineering	90.0%
8	Pharmacology & Toxicology	96.0%	19	Mathematics	90.0%
9	Agricultural Sciences	94.0%	20	Social Sciences, General	90.0%
10	Economics & Business	94.0%	21	Chemistry	86.0%
11	Geosciences	94.0%	22	Physics	84.0%

The fields are sorted in descending order by the precision. The precision for overall is 93.3% $\left(=1,026/1,100\ast 100\right)$

### Experiment on the conditions of the number of first words of the paper title

**Fig. 4:**
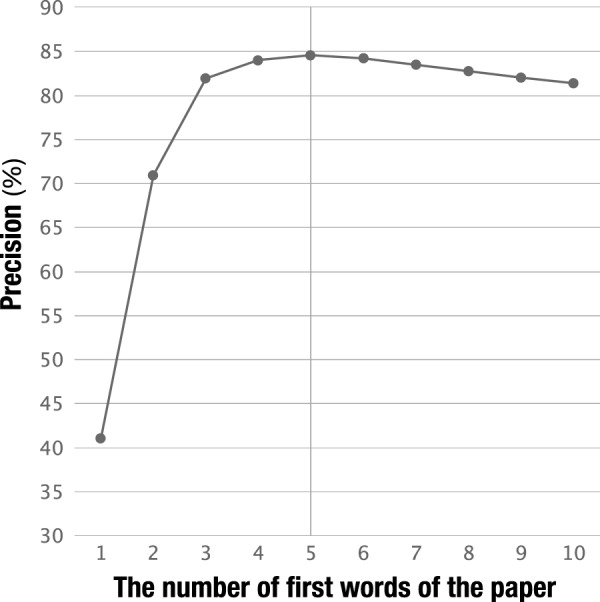
Result of precision for the combination of the full title with the first 1 to 10 words of the paper.

Fig. 4 shows the precision for each number of first words of the paper title described in Step 2-2 of the methods section. We compared the combination of the full title with the first 1 to 10 words, and the best precision was 84.6% when the first five words were employed. Hence, we used the first five words of the paper title in this study.

### Comparative analysis

In this section, we perform the comparative analysis of our dataset to the past similar dataset by Halfaker *et al*.^7^ (hereafter, “mwcite dataset”). The mwcite dataset extracted the first appearances of identifiers such as DOI, arXiv, PubMed (PMID & PMCID), and ISBN on 298 language versions of Wikipedia as of March 1, 2018. This dataset contains page ids and page titles of Wikipedia articles, revision ids and timestamps of each edit, and types and values of identifiers. Our dataset contains the first appearances of scholarly references on English Wikipedia as of March 1, 2017, and DOI names corresponding to them. As shown in Table 1, our dataset covers bibliographic metadata, research fields along with page ids and page titles of Wikipedia articles, and revision ids, editors’ information, and timestamps of each edit.

To compare with our dataset by the same condition, we extracted the records from English Wikipedia of the mwcites dataset using both DOI as the type of identifier and timestamps by March 1, 2017. DOI names of 1,020,508 in total and 721,836 in unique referenced on 229,090 pages were extracted.

Table 3 shows the results of overlapping analysis by DOI names between the two datasets. Based on the difference set, 159,952 DOI names are included only in the mwcites dataset. Out of them, 137,375 were Crossref DOIs and 20,767 were invalid DOI names. Then, 10,458 Crossref DOIs fulfill the conditions of both individual scholarly articles and identifiable research fields by Step 1-2. On the other hand, 49,235 Crossref DOIs fulfilling these conditions are included in our dataset only.

**Table 3:** Results of overlapping analysis by DOI names between the two datasets.

Target	Difference set	Product set	Total
Mwcites dataset	159,952	561,884	721,836
Percentage	22.16%	77.84%	100.0%
Our dataset	49,235	561,884	611,119
Percentage	8.10%	91.90%	100.0%

As for these 10,458 Crossref DOIs above, we took 50 random samples of the sets of DOI names, page ids, and revision ids. As a result of checking diffs between the revision ids and the previous revisions manually by the first author, they were classified into the following cases: (1) 28 cases were not written as a hyperlink but just as text (e.g., “10.1525/jps.2011.XL.2.43”), (2) 19 cases were written not as a DOI link (e.g., 10.1525/jps.2011.XL.2.43) but a hyperlink to publisher’s content (e.g., https://www.jstor.org/stable/10.1525/jps.2011.XL.2.43), (3) 2 cases were the text commented out and not displayed on the article, (4) 1 case was using Wikipedia’s template but not displayed as a DOI link due to typo.

These results show that most of the Crossref DOIs included only the mwcites dataset were not the target of this study. 10,458 Crossref DOIs fulfill the conditions of both individual scholarly articles and identifiable research fields, but they would not be written as DOI links. Apart from these cases, 49,235 Crossref DOIs fulfilling the conditions above were included in only our dataset. These gaps are interpreted as a difference in the setting of the scope. There are some differences in the setting of the scope of the target, however these two datasets contain the common DOI links at high rates, 77.84% of the mwcites dataset and 91.90% of our dataset.

Table 4 illustrates the results of overlapping analysis by the pairs of DOI names and page ids between the two datasets. Based on the product set, 814,326 pairs are common, accounting for 79.90% and 88.29% of the mwcites dataset and our dataset, respectively. Table 5 shows the results of the comparison of timestamps of these common pairs. The timestamps in both datasets were the same in 415,272 (51.0%) cases of all. For others, the timestamps in our dataset were older than those in the mwcites dataset in 399,008 (49.0%) cases, and the reverse cases were 46 (0.01%). As for the 399,054 cases that the timestamps between the two datasets were not equal, we calculated the time lag for them in days. The average was 723.2, the median was 1.5, and the standard deviation was 811.0. Based on the precision of the proposed method in this study, these gaps in timestamps show that the proposed method made advancements from the past work in identifying correct first appearances of the scholarly references.

**Table 4:** Results of overlapping analysis by the pairs of DOI names and page ids between the two datasets.

Target	Difference set	Product set	Total
Mwcites dataset	206,182	814,326	1,020,508
Percentage	20.20%	79.80%	100.0%
Our dataset	107,979	814,326	922,305
Percentage	11.71%	88.29%	100.0%

**Table 5:** Results of comparison of timestamps between the two datasets.

Group	# of records	Percentage
Our dataset = mwcites dataset	415,272	51.00%
Our dataset < mwcites dataset	399,008	49.00%
Our dataset > mwcites dataset	46	0.01%
Overall	814,326	100.0%

“Our dataset = mwcites dataset” refers to the cases where two timestamps are the same. “Our dataset < mwcites dataset” refers to the cases where the timestamps on our dataset are older than those on mwcites dataset, “Our dataset > mwcites dataset” refers to the reverse cases.

Finally, we summarize the advantages of each dataset. The mwcites dataset covers a lot of language versions of Wikipedia and multiple identifiers other than DOI names. It would be suitable for those who analyze the various and large-scale identifiers on Wikipedia or compare them across Wikipedias. On the other hand, it would be unsuitable for analyzing who and when added the original references to the page. Our dataset is focused on individual scholarly articles associated with the ESI categories referenced on English Wikipedia, hence, it would be useful for comparing them across research fields. Also, our dataset is suitable for analyzing who and when added the original references to the page.

### Basic statistics

Table 6 shows basic statistics of the dataset. On the whole, we identified the first appearances of 923,894 scholarly references (611,119 unique DOIs) in 180,795 unique pages. These references are added by 74,456 users, 63 bots, and 37,748 IP editors. With regard to research fields, “clinical medicine,” “molecular biology & genetics,” and “multidisciplinary” are top 3 for the number of total DOIs and exceed 100,000.

**Table 6:** Basic statistics of the dataset.

Research field	# of total	# of unique	# of unique	# of unique editors		
	DOIs	DOIs	pages	User	Bot	IP
Economics & Business	11,525	8,966	5,131	3,363	7	913
Social Sciences, General	55,407	41,232	27,431	14,744	17	3,301
Psychiatry/Psychology	40,016	30,250	8,761	8,640	12	2,299
Immunology	17,837	13,468	7,011	3,506	10	933
Molecular Biology & Genetics	105,668	52,770	27,288	9,546	17	2,914
Plant & Animal Science	70,433	43,143	33,487	8,415	18	2,914
Microbiology	19,923	14,644	10,667	3,256	14	916
Biology & Biochemistry	90,544	61,232	31,654	11,533	22	3,634
Clinical Medicine	124,417	95,944	32,882	18,216	26	6,589
Pharmacology & Toxicology	24,914	18,307	11,440	4,878	11	1,671
Agricultural Sciences	8,460	6,646	4,326	2,565	10	661
Multidisciplinary	102,139	51,374	42,388	16,847	26	5,765
Neuroscience & Behavior	42,186	32,108	12,096	7,687	10	2,587
Environment/Ecology	22,370	15,971	12,027	5,441	18	1,255
Chemistry	42,460	33,644	14,774	6,122	17	2,544
Geosciences	32,105	19,977	12,294	3,898	11	2,625
Space Science	38,543	15,203	10,848	2,344	13	963
Mathematics	19,876	15,157	8,533	2,831	15	1,009
Materials Science	5,673	4,556	2,541	1,713	7	564
Physics	26,191	19,039	9,249	5,402	16	2,127
Engineering	11,198	9,156	6,159	3,762	12	1,149
Computer Science	12,009	8,954	6,478	3,939	12	1,434
Overall	923,894	611,119	180,795	74,456	63	37,748

## Usage Notes

In this section, we present two demonstrations to illustrate the potential of our dataset.

### Top editors

Table 7 describes the top editors for the total number of references added to each research field. There are 13 distinct editors (ProteinBoxBot, David Eppstein, Materialscientist, The Vintage Feminist, Daniel-Brown, Meodipt, NotWith, RJHall, Rjwilmsi, Sasata, Smith609, V8rik, and Wilhelmina Will) in 22 research fields. Of these editors, ProteinBoxBot, David Eppstein, Materialscientist, and The Vintage Feminist are the top editors in multiple fields.

**Table 7:** Top editors for the total number of adding references on each research field.

Research field	Top editor	Type	# of references added	Percentage
Molecular Biology & Genetics	ProteinBoxBot	Bot	43,713	41.4%
Biology & Biochemistry	ProteinBoxBot	Bot	23,743	26.2%
Multidisciplinary	ProteinBoxBot	Bot	17,704	17.3%
Clinical Medicine	ProteinBoxBot	Bot	7,264	5.8%
Space Science	RJHall	User	4,958	12.9%
Plant & Animal Science	Sasata	User	2,975	4.2%
Mathematics	David Eppstein	User	2,662	13.4%
Immunology	ProteinBoxBot	Bot	2,616	14.7%
Chemistry	V8rik	User	2,480	5.8%
Neuroscience & Behavior	ProteinBoxBot	Bot	2,258	5.4%
Microbiology	Daniel-Brown	User	2,073	10.4%
Pharmacology & Toxicology	Meodipt	User	1,222	4.9%
Geosciences	Smith609	User	1,080	3.4%
Social Sciences, General	The Vintage Feminist	User	1,059	1.9%
Computer Science	David Eppstein	User	1,050	8.7%
Physics	Materialscientist	User	820	3.1%
Engineering	David Eppstein	User	608	5.4%
Materials Science	Materialscientist	User	508	9.0%
Economics & Business	The Vintage Feminist	User	435	3.8%
Agricultural Sciences	NotWith	User	341	4.0%
Psychiatry/Psychology	Rjwilmsi	User	303	0.8%
Environment/Ecology	Wilhelmina Will	User	298	1.3%
Overall	ProteinBoxBot	Bot	99,150	10.7%

Percentage means the proportion for the number of references added by the editor in the field. The fields and editors are sorted in descending order by the number of references added.

ProteinBoxBot^25,26^ is a bot that adds scholarly references related to molecular and cellular biology automatically at a large scale, and is the 1st editor in 6 out of 22 fields related to these fields. Furthermore, ProteinBoxBot added 99,150 scholarly references, which accounted for 10.7% of the total. David Eppstein is a computer scientist^27^, Materialscientist has received a Ph.D. in Physics^28^, and The Vintage Feminist has a social science with politics degree^29^. Thus, some 1st editors are researchers or domain experts in the corresponding fields.

### Time-series transitions

**Fig. 5:**
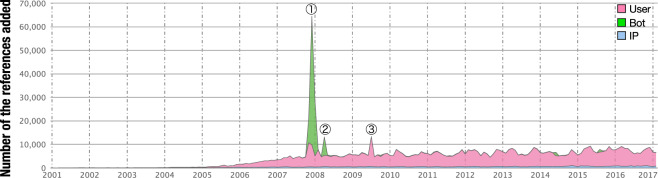
Monthly plot of the time-series transitions for the total number of references added. Each color (pink, green, and blue) refers to the type of editor who added the reference (users, bots, and IP editors, respectively).

Fig. 5 shows the monthly plot of the total number of references. The spikes seen at ①, ②, and ③ in Fig. 5 are caused by the activities of a certain editor, as shown in Table 8.

**Table 8:** Top 5 months for the total number of the references added.

#	Year and month	Total number of the references added in this term	Note
1	2007-12	64,833	ProteinBoxBot added 54,991 (84.8%) references (① in Fig. 5).
2	2008-01	28,837	ProteinBoxBot added 23,763 (82.4%) references (① in Fig. 5).
3	2007-11	21,447	ProteinBoxBot added 10,565 (49.4%) references (① in Fig. 5).
4	2009-07	13,351	Yeast2Hybrid added 8,201 (61.4%) references (③ in Fig. 5).
5	2008-04	13,224	ProteinBoxBot added 7,856 (59.4%) references (② in Fig. 5).

## Data Availability

The source code to generate the dataset in this study is available on Zenodo^30^. The code is written in Ruby. Installing ParsCit (https://github.com/knmnyn/ParsCit) is required to run this program. This code is applicable to any language version of Wikipedia. We attached sample data of the revisions on the pages “Fair trade” and “Solomon Islands” as well as identifiers referenced on these pages of English Wikipedia to enable anyone to generate a part of the dataset. To generate the full dataset, the following preprocessing is needed: (1) download the dump data of Wikipedia and apply Step 1-1 to Step 1-5 described in building the basic dataset section. (2) obtain all revisions of the pages derived from (1) by applying Step 2-1 described in building the basic dataset section, and converting them to JSON lines format. After this preprocessing, the codes corresponding to Step 2-2 above are available by just running “main.sh”. If the type of each editor is needed, Step 2-3 above should be performed.
